# Limitations of the Digestible Indispensable Amino Acid Score (DIAAS) and Choice of Statistical Reporting. Comment on “A Comparison of Dietary Protein Digestibility, Based on DIAAS Scoring, in Vegetarian and Non-Vegetarian Athletes. *Nutrients* 2019, *11*, 3106”

**DOI:** 10.3390/nu12041183

**Published:** 2020-04-23

**Authors:** Angela Genoni, Joel C Craddock, Emma F Strutt

**Affiliations:** 1 School of Medical and Health Sciences, Edith Cowan University, 270 Joondalup Drive, Joondalup 6027, Australia; 2 School of Medicine, Faculty of Science Medicine and Health, University of Wollongong, Wollongong 2500, Australia; jcc256@uowmail.edu.au; 3 Doctors for Nutrition, 6 Todd St, Port Adelaide 5015, Australia; greenstuffnutrition@gmail.com

Dear Editor,

We read with great interest the recent article by Ciuris et al. [1]; however, we feel that the authors’ conclusions suggesting vegetarian athletes may need to consume an extra 10–22 g of protein per day may be disingenuous.

The absence of fruit and vegetable food groups in the DIAAS analyses would have skewed the results, as vegetarians consume a considerable amount of these foods and, importantly, obtain a greater amount of total energy from these foods compared with non-vegetarians [2,3]. In the Adventist Health Study-2 (AHS-2), vegetarians consumed a combined total of 704.2 g of fruit and vegetables per day [2]. As fruit and vegetables can range in protein from 0.5–1.1 and 0.1–4.9 g per 100 g, respectively [4], it is likely fruit and vegetable intake would provide substantial and clinically relevant quantities of digestible protein in vegetarian diets. Furthermore, vegetarians consume more legumes, soya foods, meat analogues, nuts, seeds and wholegrains compared to non-vegetarians [2,3] which are generally poorly represented in the DIAAS calculation spreadsheet.

The majority of DIAAS modelling has been carried out in animal models using raw foodstuffs, without heat treatment [5]. It is acknowledged that cooking techniques can increase protein digestibility with the combination of fermentation and cooking increasing the digestibility of grain protein to a level near to that of meat [5,6]. In the context of exercise, DIAAS analyses do not consider the metabolic demand for protein in athletes. Physical activity can directly affect the digestibility of dietary protein, lowering the amount required to provoke anabolic effects compared to that required in sedentary states [7]. Interestingly, despite the authors’ suggestions for increased protein intake for vegetarian athletes, the current evidence shows no difference in performance outcomes between dietary groups [8].

For a cross-sectional study, it is statistically erroneous to compare strength differences between groups without adjustment for age, gender, energy intake and lean body mass. The statistical methods should have included multivariate analyses including age, energy intake and lean body mass as covariates, as these are known confounding variables [9]. The erroneous statistical methods may have led to inflated conclusions around increased lean body mass in omnivorous participants. The stratification of plant protein intake into animal protein vs. plant protein, including vegetable intake, may also provide a more accurate picture of the impact of dietary intake on muscle strength.

Considering the absence of fruit and vegetables from the DIAAS analyses, the limitations with the DIAAS and athletes, and flawed statistical reporting, it is highly likely that the additional 10–22 g of protein required daily for vegetarian athletes to achieve an intake of 1.2–1.4 g/kg/day reported by Ciuris et al. would substantially decrease or disappear completely.
